# MYB sommeliers: MYB24 drives UV response on the light side of variegated grape

**DOI:** 10.1093/plcell/koad234

**Published:** 2023-09-12

**Authors:** Carlisle Bascom

**Affiliations:** Assistant Features Editor, The Plant Cell, American Society of Plant Biologists; Natural Resources and the Environment Department, University of New Hampshire, Durham, NH 03824, USA

Plants produce myriad pigments not only to capture light for photosynthesis but also to protect their cells from damaging levels of radiation. Further, pigments such as the red-blue anthocyanins and betalains and the yellow-orange carotenoids, when produced in the flowers and fruits, contribute to attracting pollinators and seed-dispersing organisms (Tanaka et al. 2008). The berries of the grapevine (*Vitis vinifera)* are renowned for their purple (“red” and “black” grapes) and green-yellow (“white” grapes) hues. Differences in grape color largely stem from mutations in 2 transcription factors in the *Myeloblastosis* (*MYB*) gene family, *MYBA1* and *MYBA2*. MYBA1/MYBA2 function promotes the accumulation of anthocyanins in cells, and therefore null mutants produce white grapes (Kobayashi et al. 2004). In many plants, rare mutations give rise to organs that lack pigments only in some cells or sectors, resulting in a phenotype known as variegation. *Vitis vinifera “*Béquignol Noir” is a unique grape variety in which 4% of grapes from 53% of fruit clusters are variegated. In this issue of *The Plant Cell*, **Chen Zhang and colleagues** (Zhang et al. 2023) report their investigation into the pattern of pigment accumulation in these unique grapes (see Fig.). Using a combination of transcriptomics and metabolomics, the authors thoroughly characterized the stark phenotype of variegated grapes in an otherwise genetically uniform background.

**Figure.**
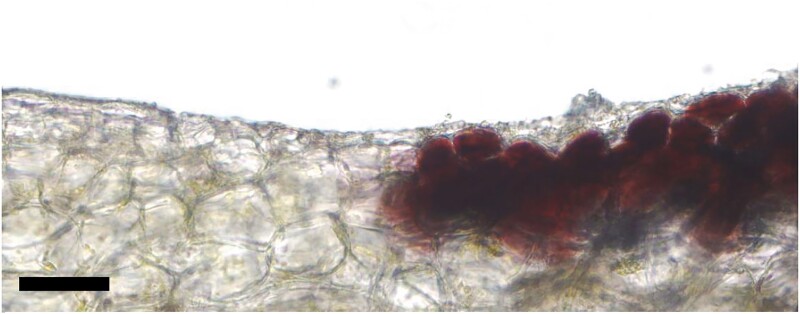
Light-microscopy cross-section of the variegation boundary in a *Vitis vinifera* cv. “Béquignol Noir” grape. Note the enrichment of anthocyanin on the right only accumulates in the outer cell layers of the fruit. Scale bar 5 *µ*m. Adapted from Zhang et al. 2023, Figure 1.

Given that the fundamental genetic mechanism underpinning “Béquignol Noir” variegation is likely the same as described previously (Kobayashi et al. 2004), the authors asked what specific pigments accumulate in the differently colored regions of variegated grapes. Further, the authors sought to elucidate the biosynthetic pathways involved and their genetic regulation.

Microarray analyses identified hundreds of differentially expressed genes between the 2 skin colors. Red regions had an abundance of genes related to anthocyanin biosynthesis. Conversely, white regions were enriched in genes related to photosynthesis and carotenoid biosynthesis. Additionally, the uncharacterized member of the R2R3-MYB transcription factor family, *MYB24,* was upregulated in white regions. Previous transcriptomic data indicated that *VviMYB24* is induced in response to UV-B radiation within the grape skin (Carbonell-Bejerano et al. 2014).

Combining available transcriptomic data with DAP-seq experiments validated via dual-luciferase assays, the authors built a model whereby MYB24 interacts with MYC2 to activate the expression of HY5 homologue (*HYH*), carotenoid isomerase 2 (*CRTISO2*), terpenoid synthase 35 (*TPS35*), *TPS09*, and another member of the MYB family, *MYBF1. HYH* and *MYBF1* expression has previously been linked to the upregulation of carotenoid/flavonol biosynthesis genes (Loyola et al. 2016; Czemmel et al. 2017). Therefore, the authors used metabolomic techniques and showed that the white-skinned region of variegated grapes are enriched in flavonol/monoterpene/carotenoid compounds. Importantly, many of these compounds also have photoprotective properties.

Taken together, the authors demonstrate that the variegated regions of “Béquignol Noir” grapes are not merely devoid of anthocyanins. In fact, in the absence of photoprotection by anthocyanins, UV radiation induces the upregulation of transcription factors that, in turn, promote the accumulation of specialized metabolites in white regions of the fruit.

This raises the question of whether this compensation occurs in variegated plants across the plant kingdom, or if there are other mechanisms of compensation. Are the metabolomic profiles between variegated fruits and variegated leaves similar? More fundamentally, do other plant tissues compensate at all? The work presented by Zhang and colleagues highlights that we have the tools available to answer such intriguing questions in non-model plant species.
